# Ultrafine MoS_2_ Nanosheets Vertically Patterned on Graphene for High-Efficient Li-Ion and Na-Ion Storage

**DOI:** 10.3389/fchem.2021.802788

**Published:** 2021-12-03

**Authors:** Chunguang Wei, Zhidong Hou, Huanhuan Sun, Jian-Gan Wang

**Affiliations:** ^1^ Shenzhen Cubic-Science Co., Ltd., Shenzhen, China; ^2^ Center for Nano Energy Materials, State Key Laboratory of Solidification Processing, School of Materials Science and Engineering, Northwestern Polytechnical University, Xi’an, China

**Keywords:** MoS_2_, graphene, heterostructure, Li-ion batteries, Na-ion batteries

## Abstract

Hierarchically two-dimensional (2D) heteroarchitecture with ultrafine MoS_2_ nanosheets vertically patterned on graphene is developed by using a facile solvothermal method. It is revealed that the strong interfacial interaction between acidic Mo precursors and graphene oxides allows for uniform and tight alignment of edge-oriented MoS_2_ nanosheets on planar graphene. The unique sheet-on-sheet architecture is of grand advantage in synergistically utilizing the highly conductive graphene and the electroactive MoS_2_, thus rendering boosted reaction kinetics and robust structural integrity for energy storage. Consequently, the heterostructured MoS_2_@graphene exhibits impressive Li/Na-ion storage properties, including high-capacity delivery and superior rate/cycling capability. The present study will provide a positive impetus on rational design of 2D metal sulfide/graphene composites as advanced electrode materials for high-efficient alkali–metal ion storage.

## Introduction

The increasing social concern on natural resources (e.g., fossil fuels) and environmental problems (e.g., climate change) has triggered extensive research activities on the development of sustainable and renewable resources of solar energy and wind power (Yang et al., 2011). Accordingly, energy storage systems such as electrochemical energy storage (EES) devices are indispensable to promote the utilization of these intermittent energy resources (Wang et al., 2015). In particular, rechargeable battery technologies, including Li-ion batteries and Na-ion batteries, stand at the forefront of the EES devices due to their high energy density (Yabuuchi et al., 2014). To meet the ever-increasing demands of energy/power densities for applications ranging from portable consumer electronics and electric vehicles to large-scale smart utility grids, it is of great necessity to improve the battery performance by developing advanced electrode materials.

Two-dimensional (2D) nanomaterials have drawn significant attention in recent years and have shown great promise in energy storage applications (Chhowalla et al., 2013; Anasori et al., 2017). Transition metal dichalcogenides (TMDs) represent a class of promising 2D nanomaterials for use in Li-ion/Na-ion batteries (Xu et al., 2014; Yun et al., 2018; Zhu et al., 2021). Among the well-studied TMDs, MoS_2_ has been considered to be a prominent candidate due to its high specific capacity and low cost (Wu et al., 2020). MoS_2_ shows analogous layered structure to graphite, which is composed of stacked S-Mo-S layers through van der Waals interactions (Liu et al., 2021). The unique layered 2D nanostructure permits easy insertion/extraction of ions along the interlayer channels, provides large surface area for energy-storage sites, and reduces the electron/ion transport distances compared with the bulk counterparts (Wang et al., 2017; Yu et al., 2017). However, the structurally stable 2H-phase of MoS_2_ suffers from low electrical conductivity and large volume change during charge/discharge processes, which collectively lead to poor rate performance and short cycle lifetime. To mitigate these shortcomings, the most effective strategy is hybridizing MoS_2_ nanostructures with conductive carbonaceous materials, such as graphene (Teng et al., 2016; Sun et al., 2020), carbon nanotubes (Chen et al., 2016; Wang et al., 2016), carbon nanofibers (Zhou et al., 2014; Wang et al., 2017), and carbon nanoboxes (Yu et al., 2015). The carbonaceous materials not only afford high-speed conductive pathways for improving the electrical conductivity of MoS_2_, but also function as flexible substrates for alleviating volume excursion, thereby enabling fast electrochemical kinetics and robust structural stability. 2D graphene (nanosheets) is of particular interest due to its large surface area, high electrical conductivity, and strong mechanical strength (Shao et al., 2015). A great deal of research activities have been made in fabricating various MoS_2_/graphene nanocomposites for high-performance Li-ion/Na-ion batteries (Geng et al., 2017; Wang et al., 2017; Pan et al., 2018; Li et al., 2019). However, the Li/Na-ion storage properties of these nanocomposites are strongly associated with their hybrid morphology. The uniform anchoring of MoS_2_ nanostructures tightly on the planar graphene could facilitate fast electrode kinetics and strong structural stability (Wang et al., 2020). Nevertheless, the negatively-charged graphene oxides are prone to be difficult to have a uniform coupling with the Mo-containing anions (e.g., MoO_4_^2−^ and Mo_7_O_24_^6−^) due to the electrostatic repulsion albeit with the assistance of cationic surfactants (Wang et al., 2013). Moreover, it is important to point out that the lithiation/sodiation of MoS_2_ starts with the intercalation of Li/Na ions into the interlayers due to the large open 2D nanochannels (Shan et al., 2016; Deng et al., 2017). That is to say, edge orientation of MoS_2_ on graphene may play a significant influence on the electrochemical performance. Therefore, it is highly desired to engineer the MoS_2_ nanosheets with preferential edge orientation and uniform aligning on the graphene to yield a maximum harvest of energy storage.

In this work, a rational strategy is developed for preparing a hierarchical 2D heteroarchitecture of the MoS_2_@graphene nanocomposite through a facile solvothermal method. The core issue is the use of acidic Mo precursors that allow for ultrafine MoS_2_ nanosheets patterned on the planar graphene surface, thus constructing a unique sheet-on-sheet nanostructure. Impressively, the unique hybrid morphology could achieve a strong synergistic effect of enlarging electrode/electrolyte interfaces, reducing ion/electron transport distances and buffering large volume change of the electrode. These electrochemical characteristics are of great advantage in enhancing the Li/Na-ion energy storage with large specific capacity, superior rate capability, and outstanding cycling durability.

## Materials and Methods

### Materials Synthesis

The preparation of MoS_2_/reduced graphene oxide (MoS_2_@rGO) was achieved by a solvothermal treatment. In specific, graphene oxide (GO) was obtained by a modified Hummers method. Then a certain amount of GO powder (20 mg) was dispersed into ultrapure water (40 ml). Subsequently, phosphomolybdic acid hydrate (100 mg) was dissolved into the GO dispersion followed by adding L-cysteine. The resulting mixture was then loaded in an autoclave and allowed to experience a solvothermal treatment of 180°C/15 h. After filtration, water rinse, and drying, black products were obtained. The as-obtained powders were finally treated by an annealing procedure of 800°C/2 h in a tube furnace under an inert N_2_ gas flow. For comparison, the Mo precursor of phosphomolybdic acid was replaced by the conventional ammonium molybdate tetrahydrate ((NH_4_)_6_Mo_7_O_24_ *·*4H_2_O, AMT) to prepare the control sample of MoS_2_@rGO-C through the similar solvothermal/annealing technique. A pure MoS_2_ sample was also synthesized by the same procedure.

### Materials Characterization

X-ray diffraction (XRD) (Cu Kα radiation) and a Raman spectrometer (Renishaw inVia) were used to identify the structural properties. The elemental information was recorded by X-ray photoelectron spectrometry (XPS) on an ESCALAB 250Xi. The thermal behavior of the composites was examined by thermalgravimetric analysis (TGA, Mettler Toledo TGA/DSC 3+). A Micromeritics ASAP 2020 was employed to analyze the N_2_ adsorption/desorption isotherm and the corresponding porous characteristics. Morphological and structural analysis was conducted by transmission electron microscopy (TEM, Talos F200X) and scanning electron microscopy (SEM).

### Electrochemical Measurements

The Li/Na-ion storage properties of the samples were evaluated based on CR 2016 coin cells, which were packed in an argon-filled glove box. A sticky slurry was prepared by dispersing 70 wt% of MoS_2_-based materials, 20 wt% of conductive carbon black, and 10 wt% of polyvinylidene fluoride in a N-methyl-2-pyrrolidinone solvent. The above slurry was spread on a copper foil, and circular discs were punched out for working electrodes. The mass loading of the active materials (MoS_2_/graphene or MoS_2_) is about 1.2–1.5 mg^−2^. Coin-type cells were packed by placing the counter electrode of Li foils, a Celgard 2,400 microporous polypropylene separator, and working electrodes together. The electrolyte was 1 M LiPF_6_ in a solvent of ethylene carbonate, dimethyl carbonate, and ethylmethyl carbonate with a volume ratio of 1:1:1. The assembly of Na-ion batteries was based on Na discs and the glass fiber filter paper as the separator. The electrolyte was 1 M NaPF_6_ in ethylene carbonate/diethyl carbonate (1:1 by volume). A CHI 660E electrochemical workstation was used to measure the cyclic voltammetry (CV) and electrochemical impedance spectra (EIS). NEWARE battery testing channels were implemented to obtain the galvanostatic charge/discharge profiles and the cycling performance at various current rates.

## Results and Discussion

**FIGURE 1:**
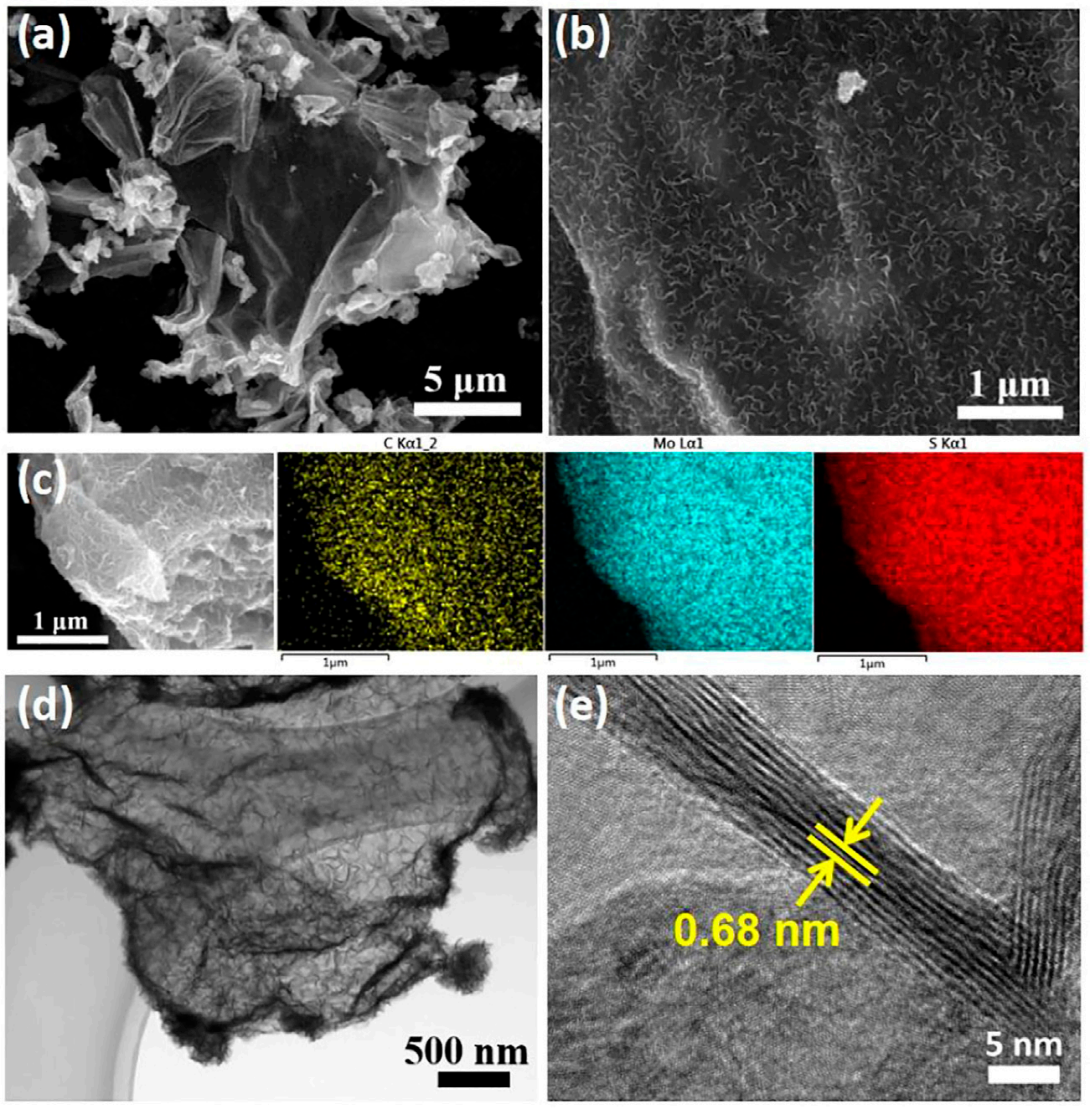
**(A,B)** SEM images of the MoS_2_@rGO composite. **(C)** EDX elemental mapping of C, Mo, and S. **(D)** TEM and **(E)** HRTEM images of MoS_2_@rGO composite.

Figure 1A presents the panoramic SEM image of the as-prepared MoS_2_@rGO nanocomposite. It is noticeable that the nanocomposite complies with the 2D morphology of graphene nanosheets without particle aggregation. A high-resolution snapshot (Figure 1B) indicates that ultrafine MoS_2_ nanosheets are tightly and uniformly patterned on the planar graphene surface, thus building a unique 2D/2D sheet-on-sheet architecture. The homogeneous anchoring of 2D MoS_2_ nanostructures onto the rGO nanosheets is validated by the even distribution of elemental C, Mo, and S from the energy-dispersive X-ray (EDX) mapping (Figure 1C). TEM imaging (Figure 1D) presents the hybrid sheet-on-sheet configuration, wherein the nanostructured MoS_2_ deposits are clearly patterning on the whole rGO nanosheet surface. The associated high-resolution TEM (HRTEM) observation (Figure 1E) reveals vertical anchoring of ultrafine MoS_2_ nanosheets with a thickness of 5–10 layers on the rGO. The lattice fringe spacing of 0.68 nm corresponds well to the distance of (002) crystal facets, which surpasses the theoretical value of MoS_2_ (0.62 nm). It is important to note that the (002)-edge-oriented and expanded MoS_2_ nanosheets are of great benefit in boosting the electrode reaction kinetics because the electrochemical lithiation/sodiation of 2D MoS_2_ generally occurs with an initial insertion of Li/Na ions in between the (002) interlayers.

**FIGURE 2:**
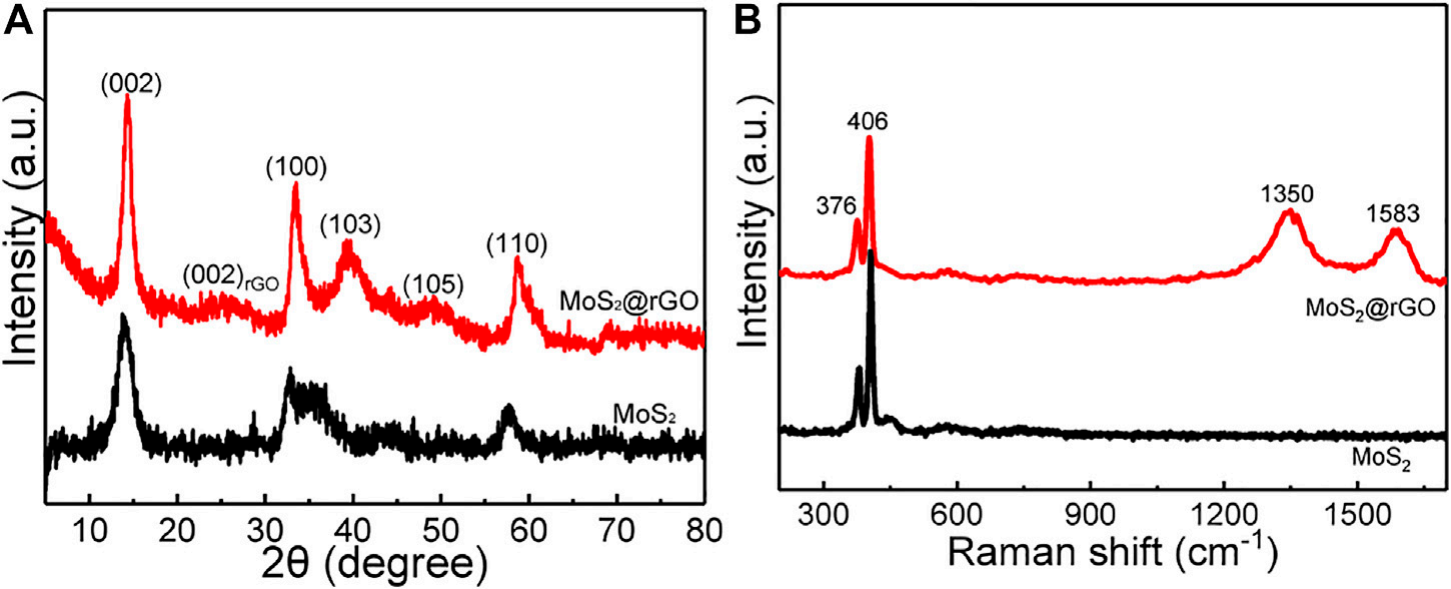
**(A)** XRD pattern. **(B)** Raman spectra of the MoS_2_@rGO composite and the pure MoS_2_.

XRD was performed to identify the crystal phase of the as-prepared samples. As shown in Figure 2A, the well-defined diffraction peaks with 2θ at around 14°, 33°, 39°, 49°, and 59° belong to the (002), (100), (103), (105), and (110) planes of 2H-type MoS_2_ hexagonal phase (JCPDS # 75–1539), respectively (Kong et al., 2014). In addition, there is a broad and low-intensity peak centered at 26°, which can be ascribed to the (002) facet of the amorphous carbon structure (Wang et al., 2021). More structural information of the samples was further examined by the Raman test (Figure 2B). Two sharp Raman peaks are noted at 406 and 376 cm^*−*1^, corresponding to the out-of-plane A_1g_ and in-plane E^1^_2g_ bands of the 2H-MoS_2_ phase, respectively (Xie et al., 2017). The Raman-sensitive carbon structure of rGO is clearly confirmed by the disordered carbon-related D band at 1350 cm^−1^ and the graphitic carbon-induced G band at 1583 cm^−1^. To determine the weight ratio of MoS_2_ in the composite, TGA was measured (Supplementary Figure S1), from which the MoS_2_ component is calculated to be ∼82 wt%.

**FIGURE 3:**
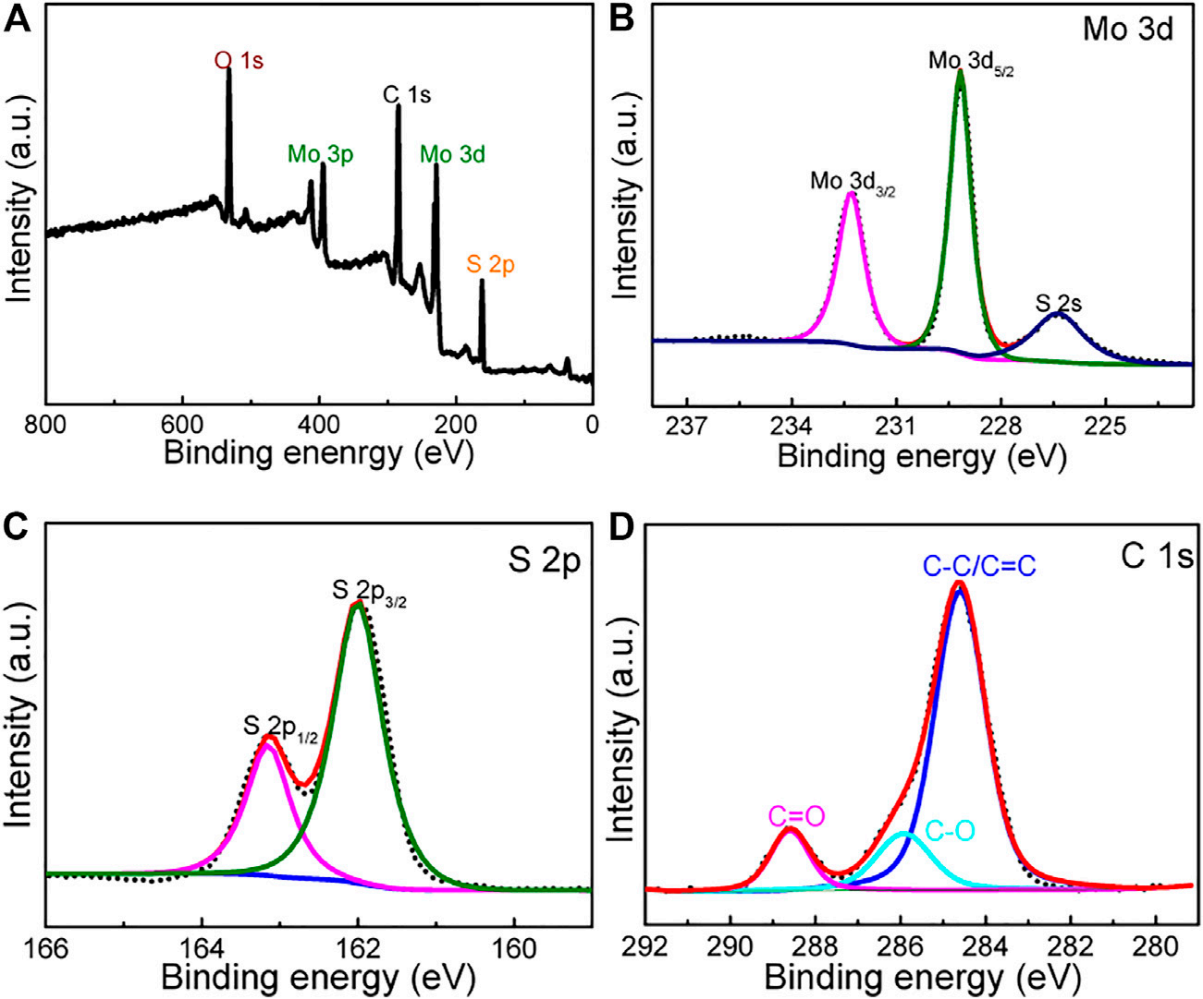
**(A)** XPS survey spectrum of the composite. **(B–D)** High-resolution XPS spectra of **(B)** Mo 3d, **(C)** S 2p, and **(D)** C 1s.

XPS measurement of the MoS_2_@rGO was carried out to probe the elemental composition and their bonding environment. The full-scan spectrum (Figure 3A) demonstrates the typical elemental signals of Mo, S, O, and C species. Figure 2C shows the high-resolution spectrum of Mo 3 days having two peaks locating at 232.3 (Mo3d_3/2_) and 229.1 eV (Mo 3d_5/2_), which agrees well with the Mo^4+^ feature of MoS_2_. Moreover, the S 2p spectrum in Figure 2D is well resolved into two components of S 2p_1/2_ (163.1 eV) and S 2p_3/2_ (161.9 eV) stemming from S^2−^ species of MoS_2_. The C1s signal (Figure 3C) is readily fitted by three parts containing C-C/C=C (284.6 eV), C-O-C (286.2 eV), and C=O (288.5 eV) bonds. This physiochemical analysis indicates the successful production of the rGO/MoS_2_ nanocomposite.

**FIGURE 4:**
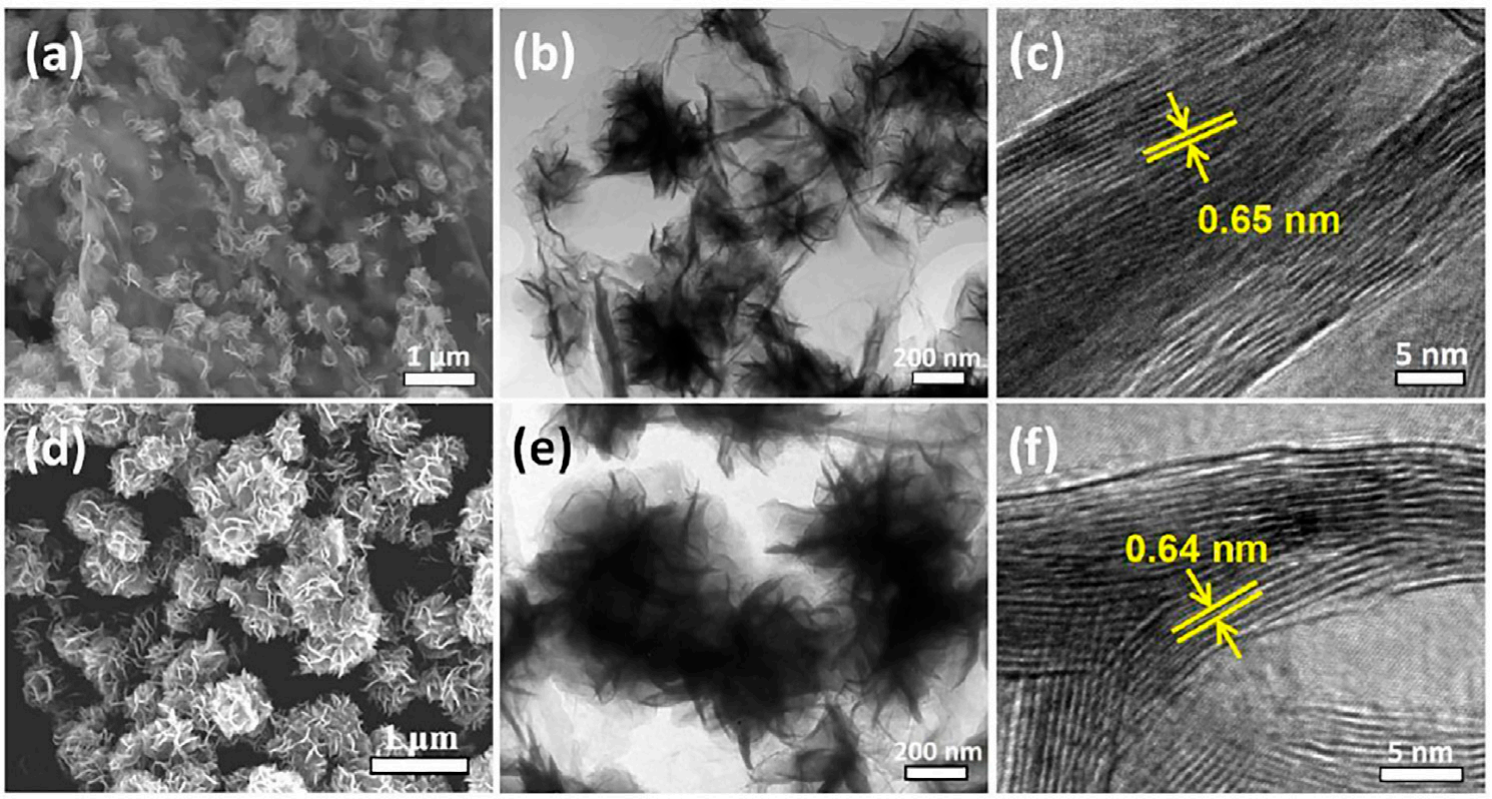
SEM, TEM, and HRTEM images of the **(A–C)** rGO/MoS_2_-C and **(D–F)** pure MoS_2_.

To highlight the structural advantage of the MoS_2_@rGO nanocomposite, a control sample of MoS_2_@rGO-C was prepared by a similar solvothermal method but using a different Mo source of ammonium molybdate ((NH_4_)_6_Mo_7_O_24_). The microstructure of the MoS_2_@rGO-C sample is observed by SEM and TEM imaging (Figures 4A,B). Notably, MoS_2_ nanoflowers are unevenly grown on the surface of rGO nanosheets, indicating a preferable growth behavior. The difference clearly demonstrates the great importance of the Mo source in tailoring the hybrid morphology. It is well-known that the surface of GO is negatively-charged induced by the functional species, such as carboxylic, hydroxyl, and phenolic groups, thus leading to weak chemical coupling with the Mo-containing anions because of electrostatic repulsion. The literature report established that the negatively charged GO surface can be modified toward a positively-charged state by reducing the pH value of the solution (Choi et al., 2015). In this work, the employment of phosphomolybdic acid as the Mo source significantly reduces the pH of the GO solution to <1, which potentially modifies the GO surface state for better coupling with the phosphomolybdic anions and thus enables the uniform formation of ultrafine MoS_2_ nanosheets. As for the ammonium molybdate or sodium molybdate used in many studies, the solution pH does not change and the MoS_2_ is prone to nucleate at some preferential sites and then grow into a large flower-like structure. For more comparison, pure MoS_2_ submicroflowers (Figures 4D,E) were prepared. It is worth to mention that the petal size of the control MoS_2_ nanosheets is much larger than that of the MoS_2_@rGO nanocomposite, and the interlayer spacing is relatively smaller (Figures 4C,F).

The Li-ion storage performance of the as-prepared samples was examined. The lithiation behavior of the MoS_2_@rGO, MoS_2_@rGO-C and pure MoS_2_ electrodes is first studied by the CV test at a sweep rate of 0.1 mV s^−1^. As shown in Figure 4A and Supplementary Figure S2, the CV curves of the three electrodes are similar, indicating the identical lithiation process induced by the electroactive MoS_2_ components (Ren et al., 2017). In specific, the broad cathodic peak at around 1.4 V in the first cycle is associated with the insertion of Li^+^ into the interlayer space of MoS_2_ to form Li_x_MoS_2_ (i.e., MoS_2_+ xLi^+^ + xe^−^ → Li_x_MoS_2_), while the following peak at ∼0.4 V corresponds to the consecutive reduction of Li_x_MoS_2_ to generate Li_2_S and metallic Mo (i.e., Li_x_MoS_2_ + (4-x)Li^+^ + (4-x)e^−^ → Mo + 2Li_2_S) and the irreversible formation of the solid-electrolyte-interface (SEI) film on the electrode surface. Upon the reverse scans, the dominant anodic peaks at about 2.3 V are reasonably attributed to the oxidation of Li_2_S to sulfur, and the 1.9 V peak is associated with the partial oxidation of metallic Mo to MoS_2_. From the second and ongoing cycles, two cathodic peaks are observed at around 2.0 and 1.4 V, which comply well with the reversible reduction of sulfur (S + 2Li^+^ + 2e → 2Li_2_S) and MoS2, respectively. It is worth to note that the CV curves are almost overlapped from the second cycle, suggesting the superb electrochemical durability and reversibility.

The cycling performance of the electrodes is examined by the galvanostatic charge–discharge test at a current density of 100 mA g^−1^. It is evident from Figure 4B and Supplementary Figure S3 that the voltage plateaus on the profiles are in good accordance with the redox couples described in the above CV analysis. The cycling comparison of the three electrodes is exhibited in Figure 4C. Remarkably, the MoS_2_@rGO electrode delivers high discharge/charge capacities of 1469/1087 mA h g^−1^, which are much higher than that of the pure MoS_2_ electrode (1100/687 mA h g^−1^). As for the control MoS_2_@rGO-C electrode, high capacities of 1428/894 mA h g^−1^ is also obtained in the first cycle, indicating the effectiveness of the conductive rGO on improving the electrochemical harvest of the electroactive MoS_2_. Moreover, in striking contrast to the MoS_2_@rGO-C (62.6%) and pure MoS_2_ electrode (62.5%), the MoS_2_@rGO electrode displays a Coulombic efficiency as high as 74% in the first cycle. The initial capacity loss is primarily stemmed from the inevitable SEI film generation and some undesirable side reactions caused by the structural defects/oxygenated carbons. The Coulombic efficiency substantially rises to 95.6% for the second cycle and preserves over 99% for the subsequent cycles, suggesting excellent reversibility of the lithiation/delithiation processes. More remarkably, there is no noticeable degradation on the specific capacity for the MoS_2_@rGO electrode, which retains at 1128 mA h g^−1^ after 100 cycles, indicating long cycling lifetime. By contrast, a significant capacity decay is observed on both the MoS_2_@rGO-C (59%) and the pure MoS_2_ (42%) electrodes upon 100-cycling. It is noted that the capacity shows a slight increase from 70 cycles, which can be ascribed to the gradual electrode activation and the reversible formation of a gel-like film (Chen et al., 2014).

**FIGURE 5:**
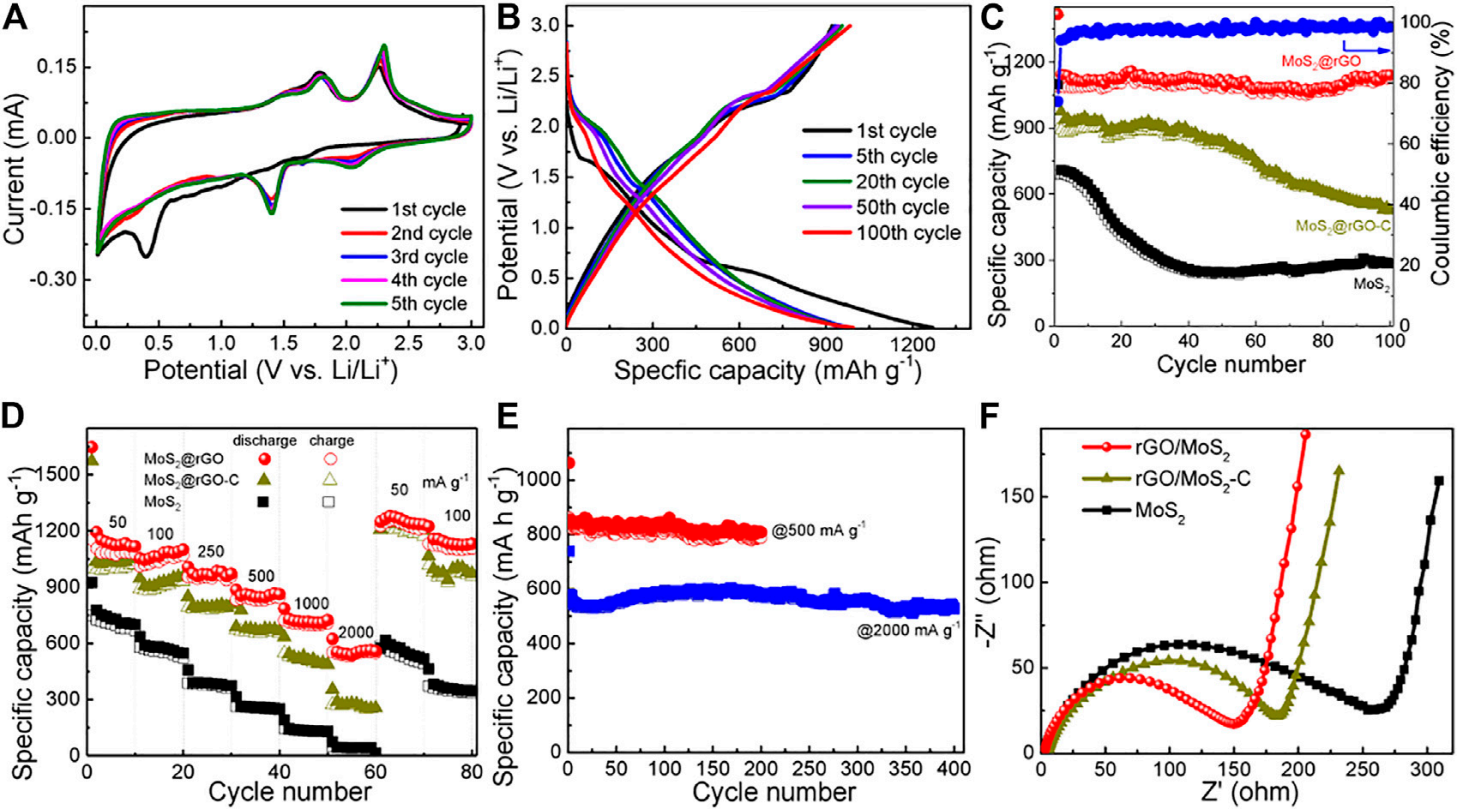
**(A)** CV curves and **(B)** galvanostatic discharge/charge profiles of the rGO/MoS_2_ electrode. **(C)** Cycling performance, **(D)** rate capability, and **(F)** Nyquist plots of the rGO/MoS_2_, rGO/MoS_2_-C, and pure MoS_2_ electrodes. **(E)** Cycling stability of the rGO/MoS_2_ electrode at high current densities of 500 and 2000 mA g^−1^.

Rate capability is an important criterion for the design of high-power LIBs (Zhu et al., 2015). Figure 4E compares the rate capability of the MoS_2_@rGO, MoS_2_@rGO-C, and pure MoS_2_ electrodes at various current densities. The hybrid rGO/MoS_2_ is capable of delivering a Li-ion storage capacity of 1122 mA h g^−1^ at a low current rate of 50 mA g^−1^. When the current rate elevates to 100, 250, 500, 1000, and 2000 mA g^−1^, the reversible capacity could keep at 1037, 936, 838, 702, and 559 mA h g^−1^, respectively. For comparison, pure MoS_2_ is almost deprived of Li-ion storage capability at the high current rate of 2000 mA g^−1^ (43 mA h g^−1^), while the control rGO/MoS_2_-C electrode merely maintains at 268 mA h g^−1^ at this current rate. This comparison clearly demonstrates the superior rate performance of the rGO/MoS_2_ electrode. Moreover, the hybrid electrode could recover the original capacity level when the current density is reduced back to 50 and 100 mA g^−1^, again suggesting that the electrode is highly reversible and durable. The prominent cycling stability of MoS_2_@rGO is further validated by another 2 cells tested at high current densities of 500 and 2000 mA g^−1^. As shown in Figure 5E, high and stable specific capacities of 759 and 542 mA h g^−1^ are sustained after 200 cycles at 500 mA g^−1^ and 400 cycles at 2000 mA g^−1^. Compared to the MoS_2_@rGO-C electrode, the rGO/MoS_2_ electrode shows substantial improvement in the cycling stability and high-rate performance. This suggests the significance of a uniform pattern and nanoscaled size of MoS_2_ on the rGO nanosheets. It is believed that the unique heterostructure holds great advantage on reducing the electron/ion transport pathways and maintaining the hybrid structural integrity. EIS analysis is carried out to gain an in-depth understanding of the electrode kinetics. Figure 5F displays the resulting Nyquist plots, in which the semicircle diameter represents the charge transfer resistance (R_ct_). As expected, compared with the rGO/MoS_2_-C (185 Ω) and pure MoS_2_ (266 Ω) electrodes, a smaller R_ct_ is noted for the rGO/MoS_2_ electrode (151 Ω). The reduced R_ct_ value implies an improved reaction kinetics, which is desirable for high power delivery.

**FIGURE 6:**
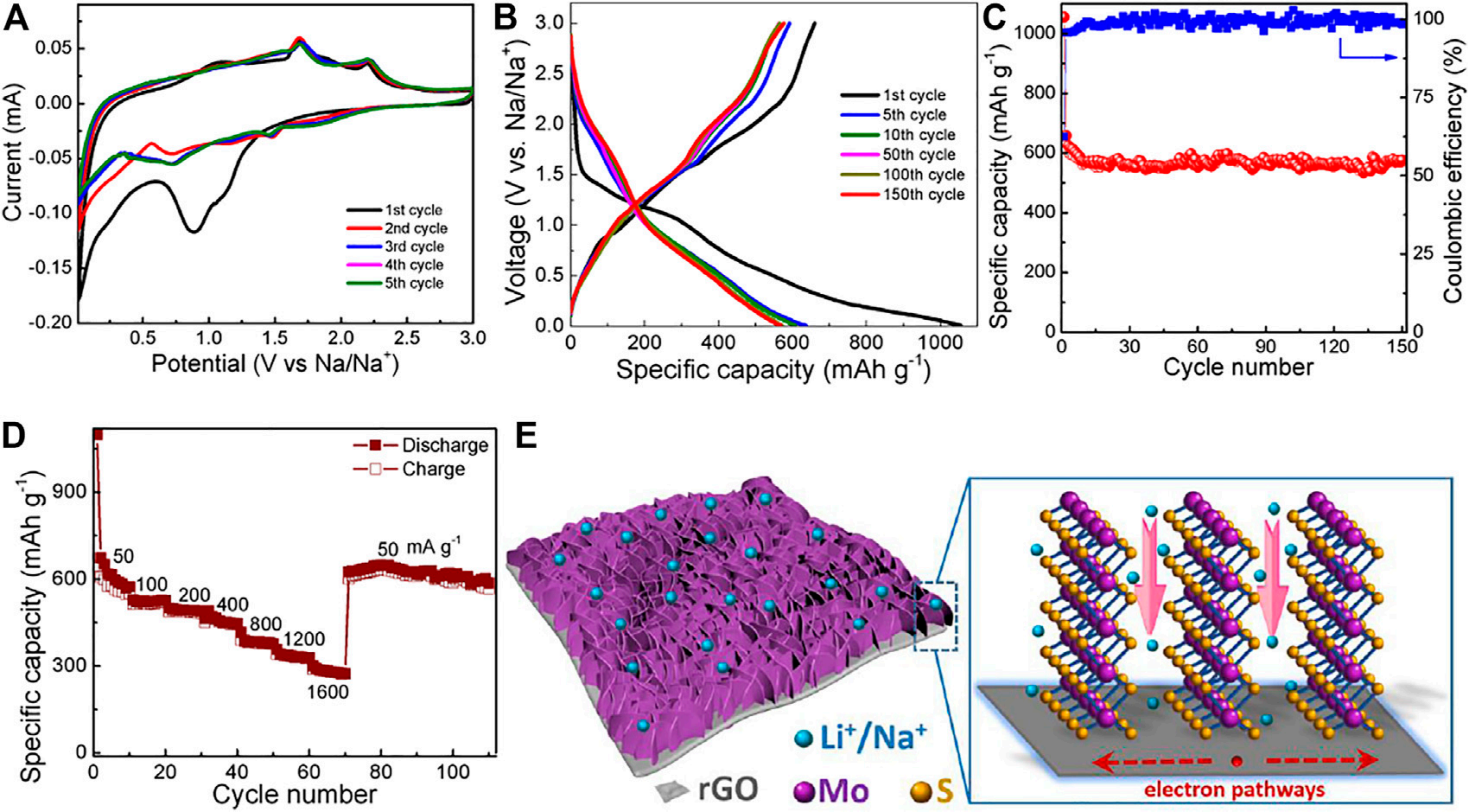
**(A)** CV, **(B)** charge/discharge curves, **(C)** cycling performance, and **(D)** rate capability of the MoS_2_@rGO electrode for SIBs. **(E)** Energy storage characteristics of MoS_2_@rGO for LIBs/SIBs.

Sodium-ion batteries (SIBs) have gained intense research activities as a promising alternative to the LIBs due to their low cost and earth-abundance of sodium in nature (Wang et al., 2018; Wu et al., 2020). Since the two battery systems share similar energy storage characteristics, it is, hence, anticipated that the unique heterostructure of MoS_2_@rGO is also of grand benefit for Na-ion storage (Wu et al., 2021). To validate our hypothesis, the Na-ion storage properties of the MoS_2_@rGO nanocomposites are examined. The resulting CV curves are displayed in Figure 6A. It is observed that the rGO/MoS_2_ electrode exhibits very similar electrochemical behaviors in the SIBs and LIBs. The broad cathodic peak at around 1.0 V in the first cycle is associated with the insertion of Na ions into the interlayer of MoS_2_ nanosheets and the formation of SEI layers. The sharp peak below 0.5 V in the following deep cathodic process corresponds to the conversion reaction of MoS_2_ to form metallic Mo and Na_2_S. The cathodic peaks in the following cycles become inconspicuous, presumably due to the gradual amorphization of crystalline MoS_2_ after the first cycle (Wang et al., 2016). During the anodic process, the two peaks can be assigned to the consecutive oxidation of Mo and Na_2_S, respectively. In addition, the anodic peaks are almost overlapped, suggesting high reversibility and cycling stability of Na-ion storage in the hybrid electrode.

Figure 6B presents the galvanostatic charge/discharge curves of the electrode at a current density of 50 mA g^−1^. The voltage profiles agree well with the sodiation/desodiation reactions of the CV peaks. The hybrid electrode delivers initial discharge and charge capacities of 1056 and 660 mA h g^−1^, respectively. The huge irreversibility of the first cycle can be attributed to the formation of SEI layers and the side reactions. However, the Coulombic efficiency increases rapidly from the initial 62.5 to 95.4% (2nd cycle) and >99% in the extended cycles. Figure 6C exhibits the cycling performance. It is noted that the MoS_2_@rGO electrode sustains a high capacity retention of 88% after 150 cycles, illustrating good cycling stability. Moreover, the hybrid electrode also manifests an attractive rate capability. As shown in Figures 6A,D a high specific capacity of 283 mA h g^−1^ is retained at a large current density of 1600 mA g^−1^, revealing a good high-rate capability of the MoS_2_@rGO nanocomposite for Na-ion storage. As the current density is returned back to 50 mA g^−1^, the electrode almost recovers its initial capacity level for extended 40 cycles, confirming the long cycle life. The Na-ion storage performance of the MoS_2_@rGO composite is compared with the previously reported MoS_2_-based materials. As summarized in Supplementary Table S1, the present performance is superior or at least comparable to those documented in most studies, demonstrating the advantage of the designed MoS_2_@rGO in SIBs.

The outstanding Li/Na-ion storage performance of the MoS_2_@rGO electrode can be rationally ascribed to the unique hybrid morphology. Figure 6E schematically illustrates the energy storage characteristics of the nanocomposite for LIBs and SIBs. First, the 2D/2D nano-architecture provides sufficient surface area for enlarging the electrode/electrolyte interfaces and thus enables more active sites for Li/Na-ion storage. Second, the nanoscaled size of the MoS_2_ nanosheets could not only shorten the ion/electron transport distance but also minimize the volume strain caused by the lithiation/delithiation (or sodiation/desodiation) processes. Third, the preferential edge termination of MoS_2_ facilitates fast ion transport through the open 2D nanochannels. Last but not the least, the flexible rGO network plays a role of high-speed conductive pathways for charge collection and transfer and also serves as an elastic substrate to alleviate the repetitive volume change of MoS_2_ during cycling operation. As a result, a strong synergistic effect is achieved between the two components to rendering a high specific capacity, a superior rate capability, and long-term cycling endurance.

## Conclusion

In summary, a hierarchical MoS_2_@rGO heterostructure has been successfully synthesized using a facile solvothermal process. The MoS_2_ nanosheets are uniformly and vertically patterning on the surface of graphene with preferential (002) edge orientation. The unique sheet-on-sheet nanoarchitecture could provide a sufficient number of electrochemically active sites, a reduced ion/electron transport distance, and a high-speed electrically conductive network, thereby generating a strong synergistic effect to enable enhanced electrode kinetics and high/stable electrochemical utilization of individual components. As a consequence, the MoS_2_@rGO nanocomposite manifests a high specific capacity, superior rate, and cycling performance for use in both LIBs and SIBs. The present work demonstrates the significance of rational engineering 2D architecture of metal sulfide/graphene to serve promising anode material candidates for high-efficient Li/Na-ion storage.

## Data Availability

The original contributions presented in the study are included in the article/Supplementary Material; further inquiries can be directed to the corresponding author.
