# Supplementary material for: Ultrafine MoS2 Nanosheets Vertically Patterned on Graphene for High-Efficient Li-Ion and Na-Ion Storage
Source: Front Chem. 2021 Dec 3;9:802788. doi: 10.3389/fchem.2021.802788 (PMC8677674; doi:10.3389/fchem.2021.802788)
Supplement: Supplementary file 1 [file Table1.DOCX]

Supplementary Material of

Ultrafine MoS_2_ Nanosheets Vertically Patterned on Graphene for High Efficient Li-Ion and Na-Ion Storage

**Chunguagn Wei^1^, Zhidong Hou^2^, Huanhuan Sun^2^, Jian-Gan Wang^2,*^**

^1^Shenzhen Cubic-Science Co., Ltd., Nanshan District, Shenzhen 518052, China

^2^Center for Nano Energy Materials, State Key Laboratory of Solidification Processing, School of Materials Science and Engineering, Northwestern Polytechnical University, Xi’an 710072, China

*** Correspondence:** wangjiangan@nwpu.edu.cn

**Supplementary Figure S1.** TGA curves of pure MoS_2_, rGO@MoS_2_ and rGO@MoS_2_-C.

**Supplementary Figure S2.** CV curves of (a) MoS_2_ and (b) MoS_2_@rGO-C.

**Supplementary Figure S3.** Galvanostatic discharge/charge profiles of (a) MoS_2_ and (b) MoS_2_@rGO-C.

**Supplementary Table S1.** A comparison of Na-ion storage performance of MoS_2_-based materials.

| Materials | Specific capacitance (mAh/g) | Cycling (mAh/g) | MoS_2_ content | Ref. |
| --- | --- | --- | --- | --- |
| MoS_2_/rGO | 702 (20 mA/g) | 430 (100 cycles) | 68.7 wt.% | [1] |
| MoS_2_/rGO | 305 (100 mA/g) | 305 (50 cycles) | 64 wt.% | [2] |
| MoS_2/_CNT | 539 (50 mA/g) | 504 (50 cycles) | 76.7 wt.% | [3] |
| MoS_2_/C | 475 (100 mA/g) | 404 (100 cycles) | 62.3 wt.% | [4] |
| MoS_2_/C paper | 320 (80 mA/g) | 286 (100 cycles) | 95.1 wt.% | [5] |
| MoS_2_/SWNTs | 410 (200 mA/g) | 390 (100 cycles) | 80 wt.% | [6] |
| MoS_2_/C | 600 (67 mA/g) | 520 (50 cycles) | 66.7 wt.% | [7] |
| MoS_2_/rGO | 380 (200 mA/g) | 442 (100 cycles) | 90.3 wt.% | [8] |
| MoS_2_/C | 494 (100 mA/g) | 432 (100 cycles) | 69 wt.% | [9] |
| MoS_2_/rGO | 338 (25 mA/g) | 218 (20 cycles) | 60 wt.% | [10] |
| MoS_2_/rGO | 573 (200 mA/g) | 480 (50 cycles) | 83.6 wt.% | [11] |
| MoS_2_/C | 620 (200 mA/g) | 477 (200 cycles) | 88 wt.% | [12] |
| MoS_2_/CNF | 381.7 (100mA/g) | 283.9 (600 cycles) | 83.2 wt.% | [13] |
| MoS_2_/C | 659 (200 mA/g) | 619.2 (100 cycles) | 41.2 wt.% | [14] |
| MoS_2_/C | 235 (200 mA/g) | 235 (350 cycles) | 98.7 wt.% | [15] |
| MoS_2_/graphene | 340 (50 mA/g) | 313 (200 cycles) | - | [16] |
| DP-MoS_2_ | 280 (500 mA/g) | 280 (200 cycles) | 100 wt.% | [17] |
| **MoS_2_/rGO** | **660 (50 mA/g)** | **581 (150 cycles)** | **82 wt.%** | **Our work** |

**Reference**

[1] X. Xie, Z. Ao, D. Su, J. Zhang, G. Wang, Adv. Funct. Mater. 25 (2015) 1393-1403.

[2] W. Qin, T. Chen, L. Pan, L. Niu, B. Hu, D. Li, J. Li, Z. Sun, Electrochim. Acta 153 (2015) 55-61.

[3] S. Zhang, X. Yu, H. Yu, Y. Chen, P. Gao, C. Li, C. Zhu, ACS Appl. Mater. interfaces, 6 (2014) 21880-21885.

[4] S.K. Park, J. Lee, S. Bong, B. Jang, K.D. Seong, Y. Piao, ACS appl. Mater. interfaces, 8 (2016) 19456-19465.

[5] X. Xie, T. Makaryan, M. Zhao, K.L. Van Aken, Y. Gogotsi, G. Wang, Adv. Energy Materials, 6 (2016) 1502161.

[6] Y. Liu, X. He, D. Hanlon, A. Harvey, J.N. Coleman, Y. Li, ACS Nano, 10 (2016) 8821-8828.

[7] J. Wang, C. Luo, T. Gao, A. Langrock, A.C. Mignerey, C. Wang, Small, 11 (2015) 473-481.

[8] R. Wang, S. Gao, K. Wang, M. Zhou, S. Cheng, K. Jiang, Sci. Rep. 7 (2017) 7963.

[9] Y. Lu, Q. Zhao, N. Zhang, K. Lei, F. Li, J. Chen, Adv. Func. Mater. 26 (2016) 911-918.

[10] L. David, R. Bhandavat, G. Singh, ACS Nano 8 (2014) 1759–1770.

[11] S.H. Choi, Y.N. Ko, J.-K. Lee, Y.C. Kang, Adv. Funct. Mater. 25 (2015) 1780–1788.

[12] Z.-T. Shi, W. Kang, J. Xu, Y.-W. Sun, M. Jiang, T.-W. Ng, H.-T. Xue, D.Y.W. Yu, W. Zhang, C.-S. Lee, Nano Energy 22 (2016) 27–37.

[13] X. Xiong, W. Luo, X. Hu, C. Chen, L. Qie, D. Hou, Y. Huang, Sci. Rep. 5 (2015) 9254.

[14] W. Ren, H. Zhang, C. Guan, C. Cheng, Adv. Funct. Mater. 27 (2017) 1702116.

[15] Y.-L. Ding, P. Kopold, K. Hahn, P.A.v. Aken, J. Maier, Y. Yu, Adv. Mater. 28 (2016) 7774–7782.

[16] X. Geng, Y. Jiao, Y. Han, A. Mukhopadhyay, L. Yang, H. Zhu, Adv. Funct. Mater. (2017) 1702998.

[17] J. Wu, J. Liu, J. Cui, S. Yao, M. Ihsan-Ul-Haq, N. Mubarak, E. Quattrocchi, F. Ciucci, J.-K. Kim, A, 8 (2020) 2114-2122.
